# Development of functionally relevant potency assays for monovalent and multivalent vaccines delivered by evolving technologies

**DOI:** 10.1038/s41541-022-00470-4

**Published:** 2022-05-05

**Authors:** Gautam Sanyal

**Affiliations:** 1 Vaccine Analytics, LLC, Kendall Park, NJ USA; 2 Bill and Melinda Gates Foundation, Seattle, WA USA

**Keywords:** RNA vaccines, Infectious diseases

## Abstract

A potency or potency-indicating assay is a regulatory requirement for the release of every lot of a vaccine. Potency is a critical quality attribute that is also monitored as a stability indicator of a vaccine product. In essence, a potency measurement is a test of the functional integrity of the antigen and is intended to ensure that the antigen retains immunocompetence, i.e., the ability to stimulate the desired immune response, in its final formulation. Despite its central importance, there is incomplete clarity about the definition and expectation of a potency assay. This article provides a perspective on the purpose, value, and challenges associated with potency testing for vaccines produced by new technologies. The focus is on messenger RNA vaccines in the light of experience gained with recombinant protein-based vaccines, which offer the opportunity to directly correlate in vitro antigenicity with in vivo immunogenicity. The challenges with developing immunologically relevant in vitro assays are discussed especially for multivalent vaccine products, the importance of which has been reinforced by the ongoing emergence of SARS-CoV-2 variants of concern. Immunoassay-based release of multivalent vaccine products, such as those containing multiple antigens from different variants or serotypes of the same virus, require antibodies that are selective for each antigen and do not significantly cross-react with the others. In the absence of such exclusively specific antibodies, alternative functional assays with demonstrable correlation to immunogenicity may be acceptable. Initiatives for geographically distributed vaccine technology facilities should include establishing these assay capabilities to enable rapid delivery of vaccines globally.

## Introduction

The coronavirus disease 2019 (COVID-19) pandemic was a catalyzing event in the rapid progression of the messenger RNA (mRNA) vaccine technology to successful product delivery^1,2^. The promise of this technology to be used as a platform for the prevention of infectious diseases outbreaks has prompted global health initiatives to establish production capabilities for mRNA vaccines in low- and middle-income countries, especially in the African continent. These manufacturing initiatives must also be accompanied by analytical capabilities to ensure that the structural and functional integrity of the antigen-encoding RNA construct is maintained throughout the process steps and, ultimately, in the formulated vaccine product. A major end goal of this process is the delivery of the functionally intact mRNA construct into the cytoplasm of target cells, where translation to the corresponding protein antigen will happen. An in vitro potency assay should be able to quantitatively measure the expression of a functionally competent protein antigen, which, in turn, will lead to in vivo induction of antigen-specific antibodies capable of neutralizing an invading pathogen.

Potency, whether it is measured by an in vitro or an in vivo assay, is, by definition, a quantifiable biological response elicited by the antigen (drug substance (DS)) or antigen formulated as the vaccine product (drug product (DP)). A dose is a quantitative measure of the active content, usually the antigen (e.g., protein) or the precursor of the antigen (e.g., mRNA). Thus, potency measurements are dependent on but not the same as dose, although this distinction has often been blurred, with some rationale, for the live attenuated virus (LAV) and viral vectored vaccines.

A potency test is a quality assurance tool that is linked to the safety and efficacy of a vaccine at administered doses, although neither can be guaranteed until extensive clinical trials have been conducted. The technologies used to produce vaccines and the resulting products determine what assays are performed to measure potency. However, for all vaccines, it must be ensured that the test result reflects or is a surrogate of the immune response desired of the vaccine. In pre-clinical phases of development, immune response in selected animal models is typically evaluated. Most often, the induction of antibodies in animal sera is measured as a function of antigen doses. Furthermore, neutralization of the virus by sera containing these antibodies provides a measure of the efficacy of the antigen in the particular animal model being used. Such a serological test of antibody induction can serve as an in vivo potency test. However, in vivo methods require the sacrifice of large numbers of animals and often suffer from long turn-around times, and a lack of robustness and reproducibility. Many regulatory agencies and manufacturers prefer in vitro potency tests for lot release. As recent examples, mRNA and viral-vector-based COVID-19 vaccines were released in Europe and USA on the basis of in vitro potency assays in accordance with recommendations by the World Health Organization (WHO) and European Medicines Agency (EMA). In vitro potency assays have been developed over the years for vaccines produced by all technologies and these assays are founded on correlation with testing in in vivo models in pre-clinical studies. It should be noted, though, that there is significant regulatory resistance in some parts of the world to accepting in vitro potency assays for lot release. Additionally, In vitro potency assays may not be feasible in certain situations. For example, binding of antibodies putatively specific to immunologically relevant epitopes may not necessarily translate to neutralizing immune response in vivo. For multivalent vaccines against either different serotypes or mutational variants of the same parent pathogen, specific and selective antibodies against each antigen representing a distinct serotype or variant may not be available.

Antibody-based immunoassays offer the possibility of correlating in vitro potency of an antigen (also called antigenicity) with in vivo immunogenicity, especially when an immune response is predominantly driven by the humoral mechanism. Recombinant protein-based subunit and virus-like particle (VLP) vaccines have been most well-studied in this regard. The mRNA vaccine platform relies on the intracellular expression of the protein antigen of interest. Therefore, lessons learned from the development of potency immunoassays for protein vaccines are applicable. However, additional steps are required, including transfection of suitable cells, to enable expression of the protein encoded by the mRNA construct, before an immunoassay is performed to measure potency. Similarly, expression of the transgene, encoding for a protein antigen, is also a prerequisite for potency assays for vaccines produced by the viral vector technology. In this article, potency and potency-determining attributes of mRNA vaccines will be primarily discussed. Additionally, examples of potency assays from recombinant protein-based vaccines and further development opportunities in this area will be discussed as foundational information that may be applied to advance the characterization of mRNA vaccines. The working antigen in cells in both cases is one or more proteins. There is another similarity between these two classes of vaccines. Both typically require adjuvants in the DP formulations. Recombinant subunit protein and VLP vaccines typically require adjuvants to cross the threshold immune stimulation needed for protective immunity. The most widely used delivery vehicles for mRNA vaccines are lipid nanoparticles (LNP), which, in addition to keeping the mRNA stable and helping to transfect target cells, indirectly provide adjuvant effects via the constituent lipids.

With ever-growing numbers of disease-causing serotypes and mutational variations (variants) of a parent virus as well new or emerging viruses, the design of multivalent and combination vaccines has become increasingly important in managing the number of required vaccinations and vaccine compliance. This increases the challenges of determining the identity, integrity, and potency of each component enormously. Development of potency assays for multivalent vaccines in the final product (DP) can be particularly challenging when specific and selective antibodies against each of a set of closely related antigens may not be available. These challenges, especially for multivalent mRNA-based vaccines, will be discussed.

## Recombinant protein-based vaccines

Among different vaccine technology platforms, recombinant protein antigen-based vaccines provide the shortest and most direct paths to testing of potency, i.e., immunocompetence. Unlike mRNA and transgene-carrying viral vectored vaccines, protein subunit or VLP vaccines do not require the cell-based or cell-free expression of the antigen for measurement of potency. Furthermore, unlike LAV vaccines, no extrapolation between infectivity and immunocompetence needs to be made. However, for an immunoassay, a selective and specific antibody against each antigen is needed. Antigens with multiple epitopes need to be characterized as to which epitopes are responsible for generating neutralizing immune responses. In vivo induction of antibodies followed by virus or pseudovirus neutralization by sera of vaccinated animals are typically performed in non-clinical studies, which can serve to provide a correlation between in vitro and in vivo potency.

In vitro potency assays, employing enzyme-linked immunosorbent assay (ELISA), have been accepted by US and European regulatory agencies for VLP vaccines against hepatitis B and human papillomavirus (HPV). These vaccines contain, respectively, VLPs composed of hepatitis B surface antigen (HBsAg) and HPV L1 proteins. In vitro potency assays have been extensively correlated with in vivo measurements of neutralizing antibody induction by the antigens^3,4^. Development of in vitro relative potency (IVRP) assays for each antigen of a 4-valent and a 9-valent HPV vaccines required the development and characterization of monoclonal antibodies (mAbs) specific for each antigen representing one of the serotypes^5,6^.

Analytical and biophysical methods can be used to characterize the primary, secondary, tertiary, and quaternary structure and folded conformation of the epitopes in the antigen that the antibodies bind to. These data provide critical insights into the structural and functional integrity of the antigen. They are not necessarily required for but support lot release. These characterization methods include LC-MS (liquid chromatography coupled to mass spectrometry for detection) based peptide mapping of an epitope, relatively low-resolution spectroscopic methods such as circular dichroism, Fourier transform infrared (FTIR), and intrinsic (tryptophan) and extrinsic fluorescence probing. Higher-resolution methods may include two-dimensional nuclear magnetic resonance (NMR) spectroscopy and cryo-electron microscopy (cryo-EM). In addition, protein aggregation beyond the level of desired quaternary structure can affect potency and this is studied by light scattering techniques. These kinds of characterization experiments can be typically carried out and interpreted more readily for soluble protein antigens (including subunits and VLPs) than with live attenuated or inactivated viruses. Such orthogonal characterization assays are expected by regulatory agencies for protein antigens.

A good potency assay provides a functional reflection of the structural integrity of an antigen. However, additional critical quality attribute (CQA)-based characterization, as mentioned above, provides the structural basis of potency and helps to understand the reasons for changes in potency at a molecular level. For example, physical aggregation of HPV 11 and 16 L1 proteins beyond well-defined quaternary VLP structures, as measured by dynamic light scattering (DLS), was directly correlated to the loss of in vitro and in vivo potency of these antigens^7^.

One way to ensure that the potency assay is truly reflecting functionality is to generate vaccine samples of varying potency. Sub-potent samples can be created by heat treatment or exposure to ultraviolet radiation^8^. The ability to discriminate potency differences in a titratable fashion allows the potency assay to be used as a stability-indicating test.

## Potency assays for vaccine products containing adjuvants

Protein subunit and VLP vaccines typically require adjuvants to deliver protective immunity. Aluminum salts such as aluminum hydroxide, aluminum phosphate, and amorphous aluminum hydroxyphosphate sulfate (AAHS) are among the oldest and most widely used adjuvants. These aluminum-based adjuvants stimulate primarily a TH2 response and were used for the first recombinant protein-based VLP vaccines to be approved, Recombivax and Engerix, both against hepatitis B. AAHS is also used in Gardasil and Gardasil-9, vaccines against 4 and 9 serotypes of HPV. Immunostimulatory effects of an adjuvant cannot be assessed by a purely In vitro potency assay that is devoid of a functionally alive system. The primary purpose of potency assays on DP containing antigen–adjuvant combinations is to determine if antigenicity or immunocompetence of the antigen is compromised by interaction with the adjuvant. For example, the antigenic site(s) or epitope(s) may be masked by a high affinity physical or chemical association with the adjuvant. This would prevent antibody binding in a potency immunoassay and could have a negative impact on induction of antibody response in vivo. Such interactions may also affect the storage stability of an adjuvant-containing DP. In this context, the stability of 4-valent Gardasil adsorbed on AAHS was evaluated by an IVRP assay (and, in parallel, by other biophysical methods)^9^. Gardasil DP was found to have excellent long-term stability in the adjuvant-adsorbed state, with a half-life exceeding 130 months at 4 °C and 18 months at 37 °C^9^. IVRP was carried out after desorption of the antigens off the aluminum adjuvant using citrate–phosphate buffer. Another mAb-based potency assay method was published more recently in which desorption was not required. HPV 6 L1 protein adsorbed on AAHS was probed with a fluorescently labeled antigen-specific mAb, and the binding affinity of the antigen for the mAb was determined using high content fluorescent imaging^10^. In addition, the interactions between two non-overlapping epitopes on the antigen were simultaneously probed using two different mAbs, each carrying a different fluorescent label. This approach may be useful for developing potency assays for multivalent vaccines, depending on the availability of selective and specific mAb against each antigen. In a recent study of the aluminum hydroxide-adsorbed trivalent non-replicating rotavirus vaccine candidate, an inhibition ELISA method was developed to quantify each of the three slightly different peptide antigens from three serotypes of the virion (VPs) fused to the P2 epitope of tetanus toxoid^11^. Interestingly, small quantitative differences were apparently observed between non-adsorbed and adsorbed antigens. This was reasonably hypothesized to result from reduced availability of VP epitopes, after adsorption on aluminum hydroxide, for binding to mAbs.

During clinical development of a respiratory syncytial virus (RSV) post-fusion, soluble, fusion protein (RSVsF) based vaccine containing synthetic glucopyranosyl lipid A (GLA) in squalene emulsion (GLA-SE) adjuvant, similar biophysical characterization was carried out. The potency of the antigen was determined by an in vitro sandwich ELISA, which involved mAb probing of two important RSVsF epitopes, A and C, against each of which specific mAbs had been developed^8^. GLA-SE was found not to interfere with this assay. Analytical methods for quantitative analysis of the adjuvant components in the formulated vaccine were developed^12^.

A spike protein (S-protein) trimer-based antigen (NVx-CoV 2373) combined with Matrix-M (a saponin adjuvant) has recently been developed as a highly efficacious vaccine against severe acute respiratory syndrome coronavirus 2 (SARS)-CoV-2 including a few variants of concern (VOCs)^13,14^. Biophysical characterization of NVX-CoV 2373 included cryo-EM, which indicated structural integrity of the S-protein trimers in presence of Matrix-M^15^. ELISA and Octet / Bio-Layer Interferometry (BLI) demonstrated high-affinity mAb binding and, thereby, structural and functional integrity of the critical antigenic site in NVX-CoV 2373. These two methods were apparently used for potency assays of DS and DP. It is not clear if the presence of Matrix M1 in the DP required any modification of the assays to avoid potential interference. A recombinant S-protein-based vaccine adjuvanted with ASO3 in the DP has also entered Phase 3 clinical trials, and similar considerations for potency assay apply (https://www.sanofi.com/en/our-covid-19-vaccine-candidates/phase-3-clinical-trial-for-COVID19-recombinant-protein-vaccine-candidate).

In vitro systems have been developed to probe immunostimulatory effects, such as cytokine production, of TLR4 agonists in various formulations. However, it may not be possible to accurately predict in vivo immune responses induced by an adjuvant in an in vitro assay, which does not include a live immune system^16^. A potency assay on a DP, which includes an adjuvant and other excipients, can detect any physical–chemical impact of the adjuvant (or excipients) on the antigen. A difference in potency between unformulated bulk vaccine (DS) and DP may also be caused by a physical interference in the assay with no biological relevance. This may be probed by an independent structural or functional test.

Shingrix. a vaccine against Zoster, consists of a lyophilized recombinant varicella zoster virus (VZV) glycoprotein E (gE) antigen that is reconstituted at the time of use with AS01 adjuvant suspension^16^. A truncated form of VZV gE is expressed in and purified from Chinese Hamster Ovary cells. The ASO1 adjuvant is composed of MPL (3-O desacyl 4′-monophosphoryl lipid A) from *Salmonella minnesot*a and QS-21 (a saponin molecule from the plant extract *Quillaja saponaria* Molina) combined in a liposomal formulation consisting of dioleoyl phosphatidylcholine (DOPC) and cholesterol in phosphate-buffered saline solution^17–19^. As the antigen and adjuvant components of Shingrix are supplied in two separate containers, lot release tests can be carried out on each component separately. MPL and QS21 in the ASO1 adjuvant have a synergistic effect in inducing antigen-specific CD4+ T cell response and antibodies, which leads to a protective immune response^20^. While the antigenicity or potency of the antigen alone is measured by an in vitro ELISA, the immunostimulatory potency of the adjuvanted vaccine product is determined using in vivo serological methods.

Observations of synergistic humoral and cell-mediated immune responses elicited by ASO1 and related dual adjuvants have prompted their use in pre-clinical and clinical development of vaccines for several infectious diseases including the recombinant chimeric protein antigen-based RTS,S vaccine against malaria^21–23^. This chimeric antigen is composed of central repeat sequences of *Plasmodium falciparum* circumsporozoite protein (CSP) and C-terminal T cell epitopes of CSP fused HBsAg. Antibodies against both components, the CSP and HBsAg, have been detected in human sera upon vaccination. ELISA for quantification of both components in the vaccine is available. Potency and immunogenicity for clinical trial vaccine DP (containing antigen and adjuvant) were, however, evaluated by in vivo measurements in mice^24^.

## mRNA vaccines

The first nucleic acid-based vaccines to be ever approved for human use are the two mRNA vaccines against COVID-19^25–28^. It is apparent that expression in transfected cells of the Spike protein (S-protein), encoded by the mRNA sequence, was quantitatively detected using flow cytometry or immunoblotting aided by a mAb against the S-protein. More details on the pre-clinical characterization of BNT162b1 and BNT162b2, published recently, have included this approach^29^. A similar approach was utilized for plasmid DNA (pDNA) vaccines that were in development for prophylaxis against other pathogens. For example, for SARS-CoV, expression of the pDNA-encoded nucleoprotein (N-protein) antigen was quantified using an N-protein-specific mAb^30^. Although pDNA must first enter the cell nucleus to undergo transcription to yield the corresponding mRNA, the final step of mRNA translation into the protein antigen in the cytoplasm is the same as for mRNA vaccines. Therefore, potency assays for pDNA and mRNA vaccines can be very similar, i.e., measurement of protein antigen expression upon transfection of eukaryotic cells. What is really determined in these potency assays is that a functionally active antigen, which is encoded by the pDNA or mRNA sequence, is indeed expressed in mammalian cells (including those of human origins, such as HEK-293 and HeLa). LNP or liposomal formulations carrying the mRNA construct in the finished DP can be directly tested in a cell-based potency assay, as the expressed protein antigen is released and accessible to the antibody. The binding of the expressed antigen to specific mAbs used for detection is a strong indication that the functionally important epitopes are conformationally preserved. This confirmation by itself does not guarantee that virus-neutralizing antibodies will be induced upon administration of the candidate pDNA- or mRNA-based vaccine. It is, however, a valid basis for the expectation that antibodies targeting the encoded antigen will be produced. During development, non-clinical animal models are typically used to determine if there is a dose-dependent correlation between antigen expression (in vitro) and antibody production (in vivo). The presence of a correlation strengthens the rationale for cell transfection-based potency assays for the lot release of nucleic acid-based vaccines.

Testing of in vitro potency and potency-determining attributes is important for evaluating process consistency, especially as manufacturing processes are scaled up and scaled out. For mRNA DS, UTR, poly-A tail and 5′-capping play critical roles in protein expression from the antigen-encoding RNA sequence. Furthermore, a critical step is the production of the LNP encapsulated DP. As an example of this last step, microfluidics channel-based production has been described in which the mRNA DS in an aqueous buffer is mixed at a controlled rate with selected lipid components leading to encapsulation of the DS into LNPs of well-defined sizes with low polydispersity^31,32^. The effect of this process needs to be determined by potency and mRNA integrity assays. After the desired parameters for LNP size and concentration are established, the microfluidics process is scaled up to produce large numbers of doses. Comparability analysis of the DPs at development and manufacturing scales includes LNP size and concentration, RNA encapsulation efficiency in LNPs, and potency of the DP in cell transfection assays. The alignment of DS and DP potencies for mRNA vaccines provides a strong indication that mRNA construct integrity is retained in the DP. Potency Assays for DS before encapsulation have used a transfection agent^29^. In fact, formulation of the mRNA into LNP facilitates cell transfection due to the presence of cationic lipids in LNP and is, thereby, expected to result in a positive impact on potency. On the other hand, loss of potency as measured by expression of the functionally active protein should trigger an investigation of mRNA integrity. As an example of this latter scenario, nucleoside modification leading to loss of mRNA translation activity in LNP formulation was detected by reverse-phase ion-paired HPLC and identified (by LC-MS/MS) as modification caused by degradation impurities from ionizable lipids^33^.

Efforts are underway to produce mRNA vaccines that would be efficacious at doses much lower than the current generation of modified non-replicating mRNA vaccines. The self-amplifying mRNA (sa-mRNA) technology is a leading example of these efforts. These exciting developments come with the challenge of quantifying both dose and potency with highly sensitive techniques. An indirect assay, correlated to potency, has been reported for an sa-mRNA based candidate zika virus vaccine^34^. This assay quantified double-stranded RNA (dsRNA) positive transfected cells by an anti-dsRNA mAb and, in this case, was performed with 100 nanograms of RNA. For quantifying the expression level of the protein antigen in transfected cells using a pan-flavivirus antibody, a higher amount of RNA (4 micrograms) had to be used.

## Potency assays as stability indicators

For all classes of vaccines, potency is an important indicator of the stability of DS and DP. Functional stability depends on structural stability, which can be independently measured by appropriate analytical tools, depending on the type of vaccine. However, as a biological and immunological activity that is linked to in vivo efficacy (at least in an animal model), in vitro potency measurements are a critical component of a vaccine stability program. For protein subunit and VLP vaccines, antibody-based immunoassays typically serve this purpose. An example of establishing whether a potency assay is a sensitive indicator of functional stability was reported for the RSVsF antigen^8^. A mechanistic understanding of the cause of an observed loss of potency, in real-time or accelerated stability studies, comes from protein degradation and aggregation analysis at all levels of structure. The use of potency assay as a stability indicator for the DP of Gardasil, a quadrivalent HPV vaccine, has been noted earlier in this article^9^.

For mRNA vaccines, the DS and DP contain the precursor of the antigen, but not the final protein antigen. Analytical measurements of RNA integrity would be direct ways of monitoring stability. Appropriate methods including HPLC coupled with charged aerosol and mass spectrometric detections have been reported^27,28^. The availability of antibodies selective for protein antigens would provide a sensitive way of measuring potency loss. mRNA vaccines are the most temperature sensitive as a vaccine class and typically require storage and transportation at ultra-low temperatures^35^. In vitro analytical assays including potency are essential tools in supporting the development of stabilizing formulations.

## Multivalent recombinant protein-based subunit and VLP vaccines

Multivalent vaccines offer prophylaxis against multiple pathogenic serotypes of a virus or against variant strains that may escape neutralizing the immune response of a vaccine developed against a specific serotype or strain initially identified. Examples of the former type are Gardasil and Gardasil-9 that offer protection against pathogenicity and carcinoma caused by 4 and 9 serotypes of HPV, respectively. The development of mAbs selective for each serotype has made it possible to determine the potency or antigenicity of each L1 protein in the combined vaccine product. In the currently approved release test for Gardasil, the potency of each antigen is measured individually. The exquisite specificity of each mAb for its target antigen would make it possible to simultaneously quantify all antigens in the product in a single multiplexed immunoassay. In fact, serological measurements of antibodies against all antigens of quadrivalent and nonavalent Gardasil in multiplexed Luminex and Chemiluminescence assays have been published^36–38^. However, lot release potency assays for both 4-valent and 9-valent Gardasil DP are carried out as singlex ELISAs, as approved by regulatory agencies.

In an interesting approach targeting multiple antigens of a single viral strain, the S-protein, membrane protein (M), and envelope (E) protein of SARS-CoV-2 have been captured in one VLP^39^. Each protein was identified in the VLP by immunoblotting. Flow cytometry, using Hep-G2 and MCP-7 cells, was used to monitor the binding of the multiprotein VLP antigen to ACE2 receptors. A plaque reduction neutralization assay was performed to demonstrate virus neutralization by sera induced in vivo after injection of the VLPs in mice. A multiplexed in vitro potency assay should be possible by using three distinct mAbs, each specific for the individual protein. While this multi-antigen vaccine is targeted against one lineage A strain of SARS-CoV-2, i.e., it is not a chimeric or combination vaccine targeting different serotypes or strains, the multivalency may help in suppressing the mutational frequency of the virus.

Bivalent vaccines that are combinations of S-proteins of SARS-CoV-2 lineage A and variant strains as well as a S-protein and N-protein combination are in pre-clinical and early clinical development^40,41^. In vitro immunoassay for potency testing is possible for the S- and N-protein combination as the two respective mAbs do not cross-react. However, selective antibodies against the S-proteins of variant strains (such as beta or B1.351) need to be developed that would not significantly cross-react with the S-protein of the lineage A strains of SARS-CoV-2.

The ability to detect and quantify each antigen will be important in the development of multivalent or combination vaccines against SARS-CoV-2 variants, and selective antibodies against each variant antigen need to be developed. A widely used mAb used for potency assays of S-protein-based vaccines is CR3022, which was originally developed for SARS-CoV. SARS-CoV-2 S-protein RBD-specific antibodies have also been developed^42^. Therapeutic mAb development efforts have focused, rightly, on providing broad coverage against multiple VOCs^43^. On the other hand, the aim of developing mAbs for characterization and potency testing of multivalent vaccines against these VOCs is to get away from cross-reactivity or to maintain differentiation by at least 10-fold.

## Multivalent mRNA vaccines

There are at least two levels of challenge with the development of a multiplexed potency assay for a DP that contains either a cocktail of multiple mRNA constructs encoding multiple proteins of variant strains or a single chimeric construct encoding these antigens^44^. First, in a multivalent vaccine, each mRNA construct must express its corresponding antigen at the same level as it does in the absence of others. Second, there should not be any interaction between the individual antigens interfering with the assay or, more importantly, affecting their potency. Additionally, just like recombinant protein subunit or VLP vaccines, mAbs with nearly 100% selectivity against each expressed antigen will be required for an immunoassay to detect and quantify each antigen. In an example of a bivalent sa-mRNA vaccine, mRNA constructs expressing nucleoprotein (N) and matrix protein (M1) were mixed in equal amounts (low nanograms) and then encapsulated in LNPs^45^. Expressions of N and M1 antigens were detected and quantified by Western blot and flow cytometry in BHK cells for each of the monocistronic (N and M1 separately) and bicistronic vectors. The percentages of dsRNA-positive cells, as detected by a dsRNA antibody, correlated with the expression level of each protein that could be detected by N and M1-specific antibodies. However, for evaluation of frequencies of dsRNA-positive cells, BHK cells were transfected by electroporation with 200 ng of RNA, an amount that could be higher than the putative sa-mRNA vaccine dose.

Several multivalent mRNA influenza vaccines are currently in development, pre-clinical and clinical, including some that have combined influenza with SARS-CoV-2^46^. The expressed antigens are distinct with selective antibodies available in each case. In vitro immunoassays for potency are, therefore, possible.

If mAbs with non-zero but low cross-antigen reactivities (10% or lower) are developed, the following strategy may be considered. For a bivalent vaccine, each of the two mAbs with low cross-reactivity could be covalently labeled with a different fluorescent label (e.g., green and red) as long as labeling does not affect its binding affinity for the expressed antigen. In a flow cytometry experiment, the intensity distribution should be resolved into predominantly green for one antigen and predominantly red for the other depending on the expression level of each of the two antigens. The same principle could be applied to multiplexed immunoassays, where both fluorescently labeled antibodies could be used simultaneously as capture antibodies

In the absence of a specific antibody against each of the antigens in a multivalent vaccine product, a combination of biophysical approaches may be applied to determine structural and functional attributes that are demonstrably correlated to their immunogenicity. Interestingly, analysis of human sera from vaccinated subjects has indicated that while mRNA vaccines developed targeting the S-protein of the initially identified and sequenced lineage A virus are significantly protective up to approximately 5 months after 2-dose vaccination against alpha, beta and delta strains, binding affinities against the variant S-proteins are weaker^2,47^. Therefore, a potential approach employing a single mAb may be to use a surface plasmon resonance (SPR)-based technology or BLI to distinguish two antigens of a bivalent vaccine by analyzing dissociation rates (off rates) that could be different by one or two orders of magnitude. If these off-rates are widely apart, appropriate sample dilution schemes may resolve the two antigens in the bivalent DP. These experiments, by themselves, will not distinguish between two epitopes on a single antigen with different affinities for the same antibody and two distinct antigens. An important component of the development of the assay should include independent measurements of affinity and dissociation rate of each antigen separately against the mAb and comparing these data with those obtained for the bivalent product. It should be noted that a widely used antibody, CR3022, has been found to have high affinities against the S-protein RBD of alpha and beta variants in addition to that of the first-sequenced Lineage A strain of SARS-CoV-2^48^. This particular mAb was originally developed for SARS-CoV and its high affinity for SARS-CoV-2 is based on a conserved epitope between the receptor-binding domains (RBD) of the S-protein of the two viruses^49^. Development and characterization of high-affinity antibodies against the RBD of SARS-CoV-2 S-protein, based on single B-cell screening and isolation of antigen-specific IgG1 memory B-cells, has been reported^50^. Such engineering efforts, while initially aimed at discovering broad-spectrum antibodies to treat COVID-19 caused by different VOCs, offer the promise of developing differentiated antibodies needed to distinguish different variants in multiplexed potency assays for multivalent SARS-CoV-2 vaccines. Analysis of memory B cells of convalescent sera from infected patients, which demonstrated the presence of neutralizing antibodies across a few VOCs with different single-site mutations in the RBD of the SARS-CoV-2 S-protein, also identified distinct epitopes in the RBD and N-terminal domain that can be potentially targeted in designing mAbs with higher variant specificities^51^.

Applications of most biophysical methods will require purification of the protein antigens expressed in transfected cells in a structurally and functionally integral form. Cell expression and purification of S2 and RBD trimer of SARS-CoV-2 from transfected cells have been reported^29^.

**Fig. 1:**
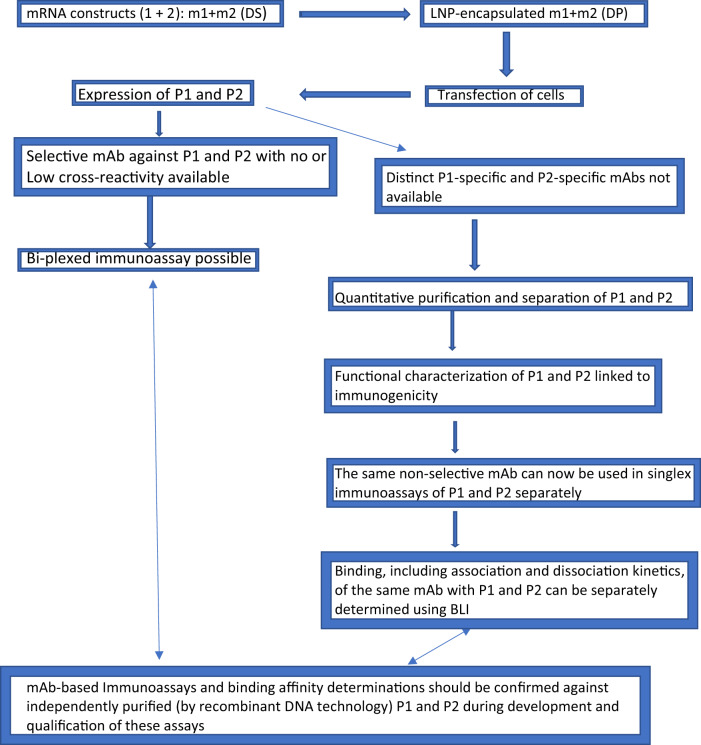
A stepwise scheme for designing potency assays for a multivalent mRNA vaccine product in final formulation using bivalent mRNA constructs as a model. m1 and m2 represent mRNA construct 1 and mRNA construct 2, respectively. P1 and P2 represent proteins encoded by m1 and m2, respectively.

The two scenarios described above, one in which a selective mAb for each antigen of a multivalent vaccine is available and the other in which this is not the case, are presented schematically in Fig. 1, which depicts putative steps in the development of potency or potency indicating assays for bivalent mRNA vaccines.

Development of potency-indicating biophysical assays will have additional challenges when potentially lower doses of more potent vaccines are delivered by new generation technologies, such as sa-mRNA. A practically feasible approach may be to transfect cells with much higher doses of each mRNA construct than the dose in the released product to extract sufficient amounts of each protein required for structural methods such as amide I FTIR spectroscopy and tryptophan fluorescence. Application of NMR spectroscopy for higher resolution structure and folding confirmation will additionally require concentrating the expressed proteins. The possibility of self-association of each expressed protein after these manipulations should be checked by light scattering techniques such as field flow fractionation coupled with multiangle light scattering and multiangle DLS, similar to their applications to recombinant subunit proteins and VLPs^52,53^. These protein antigens can individually be tested in an appropriate animal model for immunogenicity. Furthermore, animal sera could be assayed for neutralization of each of the corresponding virus variants. Once this correlation is established, one or two of the above-mentioned biophysical methods could potentially be used as a surrogate for in vitro potency assays. Furthermore, a correlation between each antigen expressed by mRNA and the corresponding variant protein purified directly (by recombinant DNA technology) should be considered at primary, secondary, and tertiary structure levels.

Developers have entered early phase clinical trials with bivalent combinations of SARS-CoV-2 mRNA vaccines, which are apparently 1:1 mixtures of the individual mRNA constructs (formulated in LNPs) targeting one or more VOCs. An example is a bivalent candidate booster vaccine, named mRNA-1273.211, which combines mRNA-1273 and mRNA-1273.351 in a single product. These mRNA constructs encode S-proteins of the initially identified lineage A strain (which the mRNA-1273 vaccine is based upon) and the B1.351 variant of SARS-CoV-2^54^.

For bivalent and multivalent vaccines, such as those including mRNA sequences encoding S-proteins of different strains of SARS-CoV-2 for which an mAb specific to each expressed antigen is not yet available, in vivo immunogenicity followed by neutralization of each variant virus (or pseudovirus) by sera of vaccinated animals will probably be required for regulatory approvals. This approach was taken for potency assessment in mice of chimeric mRNA constructs encoding different S-protein domains of SARS-CoV-2^44^. These assays will also be important for in vitro–in vivo potency correlations when specific mAbs against each antigen are available.

Assays for DP containing multivalent mRNA vaccines have additional complexities. First, each mRNA construct should be quantified to confirm the active substance content in the formulated product, e.g., in the LNP encapsulated state. An absorbance (260 nm) or a fluorescence assay (e.g., Ribogreen) will quantify total content but not be able to measure each mRNA construct individually due to non-specificity. Second, the amount of each protein antigen expressed in an appropriate cell line, transfected by the formulated DP, should be quantified. The content or dose in terms of the amount of each RNA construct in the DP will depend on the encapsulation efficiency of each mRNA construct in LNP. Advanced polymerase chain reaction (PCR) technologies such as digital droplet PCR (ddPCR) offer a powerful way of detecting mutations or variations in nucleotides between closely related DNA or RNA sequences^55,56^. This technology may be used to identify and quantify two or more RNA constructs in a multivalent mRNA DS or DP, thereby providing a measurement of the dose of each construct.

Accurate determinations of identity and integrity of the coding sequence of each mRNA, and confirmation of length and integrity of poly-A tail and UTR of each construct can be used to evaluate their fidelity or efficiency of translation to the corresponding protein antigens. A combination of these measurements can serve as a surrogate for in vitro potency assays for multivalent mRNA vaccines, especially when reinforced with a demonstration of in vivo induction of neutralizing antibodies against each variant virus in pre-clinical proof-of-concept studies. An interesting and novel concept has been recently presented for quantification and characterization of individual mRNA constructs of a multivalent mRNA vaccine^57^. Although the proposed method will not detect protein expression, it will potentially capture each construct via an oligonucleotide sequence specifically complementary to its target. Each capture oligo can carry a distinct fluorescent tag, different from the tag used to label each of the other oligos that target their complementary mRNA constructs. This approach should allow multiplexed measurements of multiple mRNA constructs in a single assay. Optimizing the capture oligos for structural in addition to sequence complementarity with their target mRNA constructs would be highly valuable. It is worth noting that in a draft guidance document prepared by WHO experts, the potential acceptability of combining multiple functional assays for potency evaluation has been suggested^58^. Ultimately, regulatory acceptance of potency assays for this new and still evolving class of vaccines will depend upon evaluation criteria and expectations, which may not be the same for all national regulatory agencies.

The formulation of the multivalent DP is an important consideration in designing the potency assay and related analysis of CQAs. For example, a chimeric, single mRNA construct composed of mRNAs encoding SARS-CoV-2 variant S-proteins has been encapsulated in an LNP formulation^44^. For a cocktail of individual mRNA constructs, the mixture of the different mRNA constructs could be encapsulated in one LNP formulation. In either case, unless and until mAbs specific to each expressed antigen are available, in vivo potency assays accompanied by neutralization of each of the variant viruses or its corresponding engineered pseudovirus will have to be pursued. A VSV-conjugated pseudovirus corresponding to a SARS-CoV-2 lineage A strain was used to test for neutralization by sera of BNT162b1 and BNT162b2 mRNA vaccinated animals^29^.

The development of multiplexed potency immunoassays for multivalent vaccines containing mRNA constructs encoding for antigens of different viruses such as influenza and SARS-CoV-2 that generate widely different neutralizing antibodies should be more straightforward, as selective and specific mAbs against each antigen are available.

## Viral vector-based vaccines

Vaccines based on viral vectors carrying transgenes that encode selected protein antigens are relatively new entries, although first deliveries by this technology predated those delivered by the mRNA technology. Both of the first two vaccines to be approved were against the Zaire strain of the Ebola virus and used replication-competent and replication-deficient vectors, respectively^59,60^. Subsequently, two replication-deficient Adenovirus vector-based vaccines against SARS-CoV-2 received emergency use authorization^61,62^. Viral vectored vaccines are expected to combine the advantages of nucleic acid-based vaccines in terms of intracellular expression of target antigens with CD4+ and CD8+ cellular immune responses stimulated by the viral element.

The vector is an attenuated or engineered form of a virus, which is different from the virus (pathogen) being targeted. Dosing has been appropriately based on viral titers (total and infectious) for both replication-competent and replication-deficient viral vectors^59–61^. Additionally, an appropriate potency assay should quantitatively measure the expression of the transgene (payload) to deliver the functionally active target antigen. This latter attribute is related to vaccine antigenicity (and, by extrapolation, to immunogenicity), while the viral vector is used to deliver the encoded antigen and potentially stimulate a cellular immune response. For the Ad26-MVA prime-boost Ebola vaccine, cell-based infectious titer assays were performed for Ad26 (prime) and MVA (boost) using quantitative PCR and flow cytometry detection, respectively. In addition, transgene expression was tracked using primers targeted at a 100-bp amplicon present in the CMV promoter of the transgene cassette (Supplementary Material in ref. ^60^). Changes in fluorescence intensity of a fluorescent probe (coupled with a quencher) annealed in the middle of the amplicon were used to quantitate viral particles that expressed the transgene in BHK-21 cells^60^. In combination, these assays served as dose and potency measurements. For an Ad26-vectored COVID-19 vaccine, expressions of proteins encoded by corresponding DNA constructs in transfected MRC-5 cells were quantified for SARS-CoV-2 S-protein, RBD, furin-cleaved S2, and furin cleavage site mutations by antibody-aided ELISA, flow cytometry and Western blot analysis^62^. A similar immunoassay approach was also used for a measles-vectored SARS-CoV-2 candidate vaccine in pre-clinical development^63^. In this study, various forms of the S-protein including pre-fusion stabilized S, full-length S, and cleaved S1 proteins were detected, after the expression of the respective genes in transfected Vero cells, by Western blot analysis, based on molecular weights. However, the distinction between dose (viral titer) and in vitro potency (transgene expression) has not always been strictly maintained for this class of vaccines. The regulatory expectation of a transgene expression-based potency assay was stated in the EMA Assessment Report on the EUA submission of one viral vectored COVID-19 vaccine, which initially included only viral titer as potency^64^.

Bivalent vaccines are also being developed using this technology and the feasibility of antibody-based immunoassays for in vitro potency testing again depends on the availability of selective antibodies against each expressed antigen. In one example, a non-replicating human Ad5 vector carrying genetic sequences coding for S- and N-proteins of SARS-CoV-2 has shown promising cell-mediated and neutralizing antibody responses^65^. As distinct and selective mAbs are available against both protein antigens, a biplexed in vitro immunoassay is feasible for potency testing.

## Closing comments

In the global efforts to rapidly develop vaccines against ever-increasing numbers of deadly pathogens, potency assays play a centrally essential role as an initial indicator of potential biological efficacy and as a validation of effective antigen design. A well-designed in vitro immunoassay can quantitatively assess the potential for a vaccine antigen to elicit the desired immune response in vivo. In addition to the pre-requisite of developing or procuring a highly selective antibody to enable a sensitive immunoassay, newer technologies such as mRNA and transgene carrying viral vectors present the challenge of demonstrating that the antigen is expressed in the cytoplasm of the target cells in structurally integral and immunologically functional form. For potency immunoassays of multivalent vaccine products, a selective, specific, and high-affinity antibody against each antigen or expressed antigen must be available. Collaborative efforts similar to those established between the Bill and Melinda Gates Foundation (BMGF), Planning Alternative Tomorrows with Hope (PATH), Coalition for Epidemics Preparedness Innovations (CEPI), and the National Institute for Biological Standards and Control (NIBSC) to address antibody supplies for potency testing of monovalent COVID-19 vaccines can, hopefully, be progressed to help developers globally^66^. The development of analytical tools to support developers and manufacturers should also be aided by focused funding initiatives recently launched by the National Institute for Innovation in Manufacturing of Biopharmaceuticals (NIIMBL) in partnership with BMGF. Rapidly emerging variants of SARS-CoV-2 during this pandemic have once again demonstrated the need for accelerated development of multivalent vaccines to keep the number of required vaccinations manageable and compliance among global populations at a high level. The mRNA technology appears to provide a very promising platform for speedy delivery of new vaccines including multivalent vaccines comprising either chimeric or a combination of multiple mRNA constructs. Either way, for cell-based potency immunoassays of vaccines encoding multiple antigens, the selection of appropriate cell lines would be important so that each construct is fully expressed in an immunocompetent form. The rapid development of reagents, especially selective antibodies, and global access for developers are important considerations. A geographically distributed common source of well-characterized cell lines would be helpful. A similar approach for access by all developers globally to specialized technologies such as Next Generation Sequencing and ddPCR should be considered for ensuring consistency and accuracy of results as well as cost-effective delivery of vaccines. In the area of potency and potency-determining analytical assays, this can be accomplished without compromising the proprietary ownership of a product delivered by each developer. Although ELISA and immunoblotting are still the most widely used immunoassay methods used in vaccine development and lot release, several new technologies developed in recent years have high sensitivity and multiplexing capabilities. Most of these methods have largely been used for serological detection of antibodies and cytokines^67–69^. However, in principle, these methods could be used for vaccine potency assays utilizing the same basic principle of specific antibody-antigen binding interactions. Comparative analyses between traditional immunoassays (such as ELISA and single radial immunodiffusion) and these newer techniques that offer multiplexing advantages have been reported, such as for Luminex and VaxArray^70–72^. It should be noted that immunofluorescence-based flow cytometry has been used for the potency assay of one of the two approved COVID-19 mRNA vaccines^29^. The ultimate choice of a particular immunoassay technology should depend upon several factors including the need for a multiplexed assay, ease of validation, suitability for use in a GMP lot release environment, cost of supplies and equipment, and uninterrupted global availability or access of the technology including required reagents. The relative ease of development, execution, and transfer of an assay technology is also an important consideration, especially in the context of affordable access to high-quality vaccines for all populations. To advance the cause of global vaccine equity and to minimize long-haul transportations of relatively less stable vaccines (e.g., mRNA-based), initiatives by philanthropic organizations as well as pharmaceutical companies have been launched to build geographically distributed manufacturing capabilities^73–75^. These manufacturing capabilities must also be complemented with at-site analytical testing of quality attributes including robust and rapid turn-around potency assays that can monitor scalability as well as improvements in process and formulation.

In addition to antibody-mediated protection, T cell-mediated immunity (CMI) is an important component of protection by many vaccines, including, possibly the mRNA and viral-vectored vaccines that have been delivered against SARS-CoV-2. However, CMI assays are not typically used for lot release, although these assays are performed as serological markers of immune responses in non-clinical animal models and in clinical (human) sera after vaccination^76^. For cytotoxic T cell-inducing vaccines, ex vivo and cell culture-based ELISPOT as well as flow cytometry can be further developed^77,78^. While adaptation as lot release potency tests may be challenging, these assays can provide important supportive data on the immunocompetence of vaccines, especially for those that utilize CMI as a major mechanism for efficacy.

## Data Availability

Data sharing is not applicable to this article as no original data were generated. The perspective presented is based on the information and data published earlier, which have been cited in this article.

## Code availability

No custom code or mathematical algorithm was used in this article.
